# Observation of the quantum Hall effect in δ-doped SrTiO_3_

**DOI:** 10.1038/ncomms11631

**Published:** 2016-05-27

**Authors:** Y. Matsubara, K. S. Takahashi, M. S. Bahramy, Y. Kozuka, D. Maryenko, J. Falson, A. Tsukazaki, Y. Tokura, M. Kawasaki

**Affiliations:** 1 RIKEN Center for Emergent Matter Science (CEMS), Wako 351-0198, Japan; 2 Institute for Materials Research, Tohoku University, Sendai 908-8577, Japan; 3 PRESTO, Japan Science and Technology Agency (JST), Chiyoda-ku, Tokyo 102-0075, Japan; 4 Department of Applied Physics and Quantum Phase Electronics Center, University of Tokyo, Tokyo 113-8656, Japan

## Abstract

The quantum Hall effect is a macroscopic quantum phenomenon in a two-dimensional electron system. The two-dimensional electron system in SrTiO_3_ has sparked a great deal of interest, mainly because of the strong electron correlation effects expected from the 3*d* orbitals. Here we report the observation of the quantum Hall effect in a dilute La-doped SrTiO_3_-two-dimensional electron system, fabricated by metal organic molecular-beam epitaxy. The quantized Hall plateaus are found to be solely stemming from the low Landau levels with even integer-filling factors, *ν*=4 and 6 without any contribution from odd *ν*'s. For *ν*=4, the corresponding plateau disappears on decreasing the carrier density. Such peculiar behaviours are proposed to be due to the crossing between the Landau levels originating from the two subbands composed of *d* orbitals with different effective masses. Our findings pave a way to explore unprecedented quantum phenomena in *d*-electron systems.

## Abstract (web summary)

Observation of quantum phenomena in correlated electron systems is challenging due to low mobility and high concentration of carriers. Here, Matsubara *et al*. report a two-dimensional electron system with high mobility-low carrier density in δ-doped SrTiO_3_, demonstrating quantum Hall effect in *d*-electron systems.

## Graphical abstract

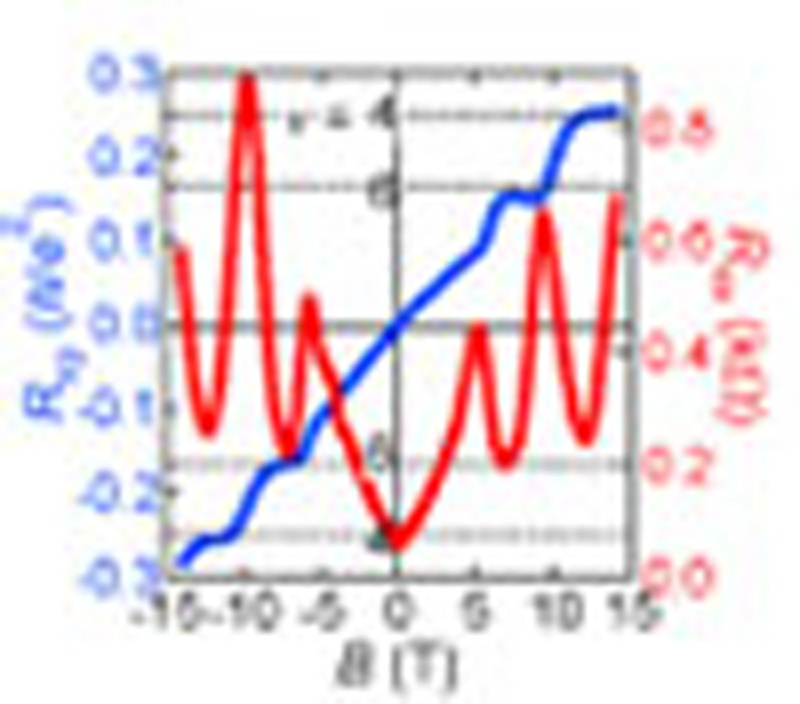

Conventional semiconductors such as Si, GaAs and ZnO are the main workhorses in the studies of integer and fractional quantum Hall effects (QHEs)^1^,^2^,^3^,^4^. The mobile carriers in these materials are located in bands composed mainly of *s* and *p* orbitals. In contrast, the conduction band of perovskite transition metal oxides such as SrTiO_3_ (STO) is composed of 3*d t*_2g_ orbitals with a strong directional anisotropy^5^. When confined into a two-dimensional (2D) environment, these states can show very interesting properties^6^,^7^,^8^. At sufficiently high carrier densities, the *t*_2g_ conduction band is quantized into a ladder of light and heavy subbands, whereas at low carrier densities resulting subbands are dominated by heavy orbitals, *d*_*yz*_/*d*_*zx*_^6^. In the latter case, electron–phonon effects combined with the many body interactions could further modify the dispersion of subbands thereby leading to formation of unusual electron liquid states^9^. Consequently, the STO-based two-dimensional electron system (2DES) can exhibit a variety of unconventional quantum effects^10^. Moreover, since STO is a widely used substrate for epitaxial growth of versatile materials with exotic properties such as high-*T*_c_ superconductivity, ferroelectricity, ferromagnetism and topological phases^11^,^12^, one can potentially incorporate a STO-based 2DES with high mobility into such systems to realize novel quantum effects.

Owing to the recent progress of thin-film growth technique, the electron mobility of three-dimensional carriers of STO has reached 53,000 cm^2^ V^−1^ s^−1^ in single crystalline films^13^, which is larger than 22,000 cm^2^ V^−1^ s^−1^ of bulk single crystals^14^. However, preserving a metallic state with reasonably high mobility has proven to be a challenge when the carriers are confined in two dimensions. While there is a large number of studies on STO-based 2DES including LaAlO_3_/STO (LAO/STO) interface and δ-doped STO^7^,^8^,^15^,^16^,^17^,^18^,^19^,^20^, the realization of the QHE in this class of systems has proven to be elusive and, thus, yet to be demonstrated. In particular, the low mobility of the doped carriers and their high concentration have hindered the successful demonstration of the QHE in STO. Any attempt to reduce the carrier density below 3 × 10^13^ cm^−2^ has turned out to result in a non-metallic state^15^. Although with the recent advancement in the growth of LAO/STO interfaces one can now realize 2DES's with a relatively low carrier density (in the range of 10^12^ cm^−2^) with maintaining a high mobility (nearly 10,000 cm^2^ V^−1^ s^−1^), it is still practically impossible to reach Landau levels with filling factors *ν*≤10 (refs 17, 18, 19, 20). The realization of QHE at low enough filling factors in an easily accessible magnetic field (∼10 T) imposes the restriction on the carrier density, which favourably should be below 1 × 10^12^ cm^−2^. At higher carrier densities, it is therefore practically only possible to observe the Shubnikov-de Haas (SdH) effect, which is of course a different quantum phenomena from the QHE.

Here we employ molecular-beam epitaxy (MBE) at a very high temperature (1,200 °C) with metal organic (MO) precursors (MOMBE)^21^ to grow STO heterostructures confining the 2DES and reach electron mobility exceeding 20,000 cm^2^ V^−1^ s^−1^ at charge-carrier densities below 1 × 10^12^ cm^−2^. With such a high mobility-low carrier density heterostructure, we can successfully reach the quantum Hall regime in STO. The 2DES shows clear signatures of the QHE but there are peculiar features; the quantization occurs only at even integer states (*ν*=4, 6) and the *ν*=4 state disappears at low carrier concentration. To elucidate the origin for those, we performed first principles calculations and found that these features can be modelled if the spin susceptibility is small compared with the Landau level broadening and the crossover of hybridized two *d* orbitals made of *d*_*yz*_ and *d*_*zx*_ are taken into account.

## Results

### Transport properties of δ-doped SrTiO_3_

**Figure 1:**
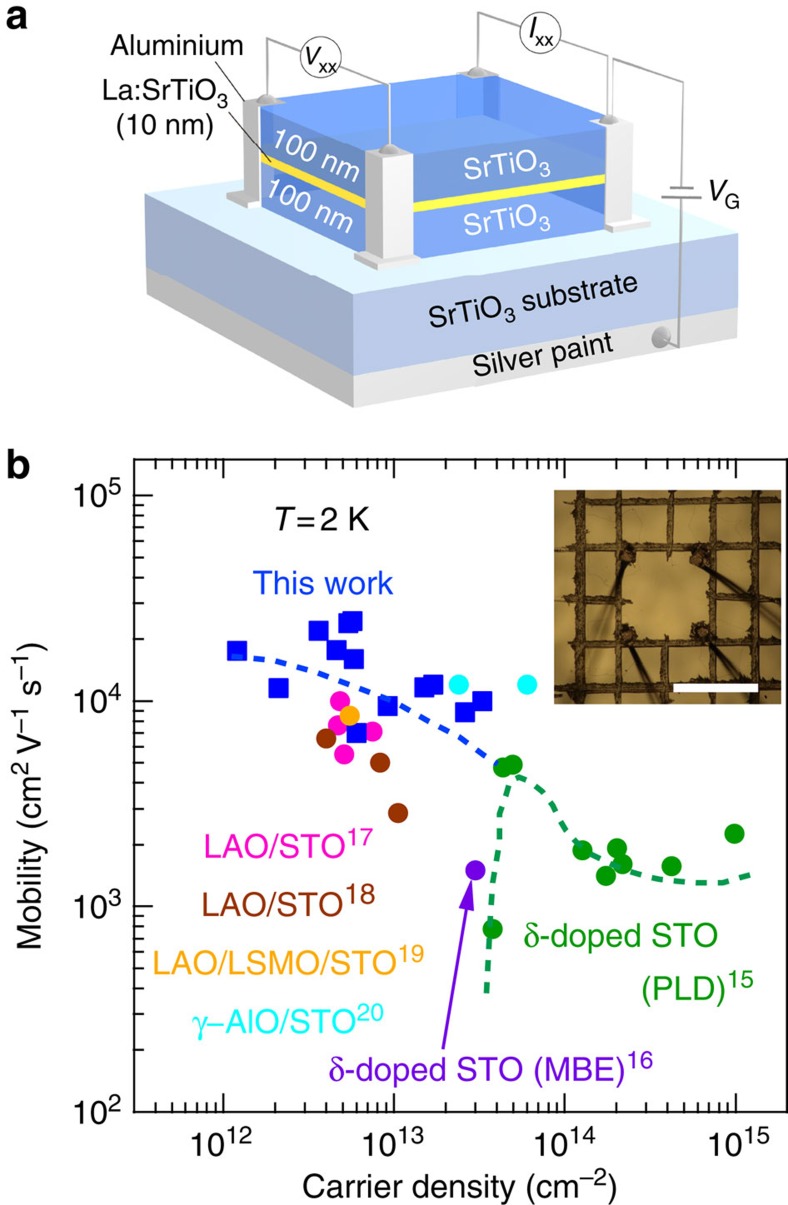
Device structure and electron mobility in δ-doped SrTiO_3_. (**a**) A sketch of a van der Pauw device with a back-gate electrode. (**b**) Electron mobility at 2 K for various samples as a function of carrier density. The data in literatures^15^,^16^,^17^,^18^,^19^,^20^ are also plotted for comparison. The inset shows an optical microscope image of the device defined by scratching. Scale bar, 500 μm.

The δ-doped STO, studied here, is composed of a 100-nm-thick bottom STO buffer layer, a 10-nm-thick STO doped with 3 × 10^19^ cm^−3^ La, and a 100-nm-thick top STO capping layer epitaxially grown on a (001) STO single-crystal substrate. The device is sketched in Fig. 1a and its corresponding optical microscope image is shown in Fig. 1b. The sample is scratched by a needle to pattern a van der Pauw type device with size 500 × 500 μm^2^. At the four edges of the scratched square, aluminium wire is ultrasonically bonded to make an Ohmic contact with the 2D layer. The longitudinal resistance *R*_*xx*_ and Hall resistance *R*_*xy*_ showing below are all raw data of one configuration in van der Pauw geometry without calculating the average with the orthogonal configuration value of *R*_*xx*_ and *R*_*xy*_. The sample is fixed with silver epoxy on a chip carrier with a metallic surface. Due to a large dielectric constant of STO at low temperature^22^, the chip carrier surface acts as a global back gate for STO-based 2DES and thus enables *in situ* tuning of carrier density. First, the as-grown samples, that is, without applying the back-gate voltage (*V*_G_), are characterized at 2 K. Figure 1b shows the relation between the ‘2D' mobility and the carrier density at 2 K for various δ-doped STO samples and STO-based heterostructures that indicate pure electron conduction. The data are picked up from previous reports showing clear evidence of 2D conduction with low carrier density such as SdH oscillations in tilted magnetic field^15^,^16^,^17^,^18^,^19^,^20^. The quantum transport measurements are performed at dilution refrigerator temperatures and in magnetic fields up to 14 T employing a low-frequency (7–9 Hz) lock-in technique with a low excitation current of 100 nA to suppress heating.

**Figure 2:**
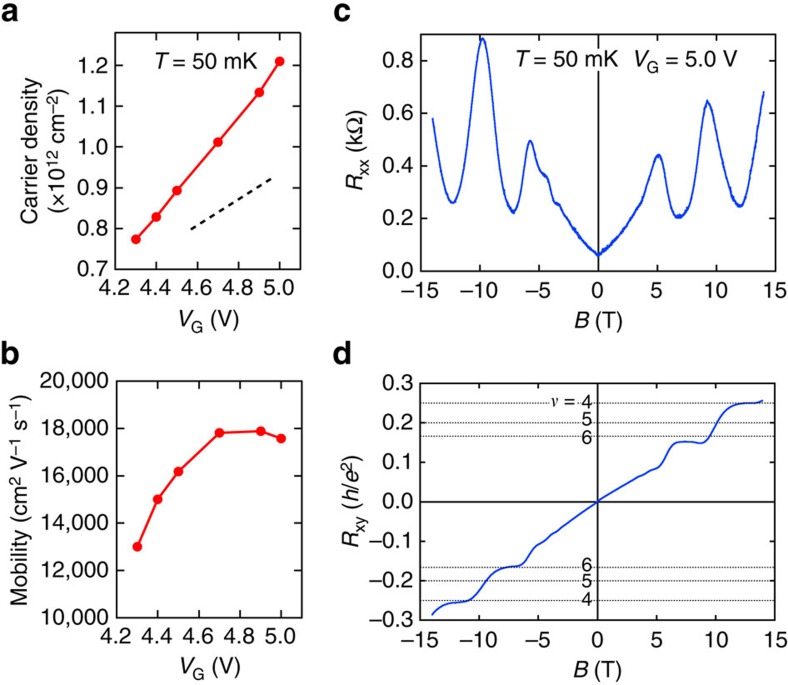
Transport properties of two-dimensional electrons in δ-doped SrTiO_3_. (**a**,**b**) Carrier density (**a**) and mobility (**b**) as a function of gate voltage *V*_G_ at 50 mK. The slope depicted as broken line, 2.8 × 10^11^ cm^−2^ V^−1^, denotes the calculated one for the carrier density against gate voltage, assuming a STO gate dielectric constant *ɛ* of 20,000. (**c**,**d**), Longitudinal resistance *R*_*xx*_ (**c**) and Hall resistance *R*_*xy*_ (**d**) versus magnetic field *B* measured at 50 mK with *V*_G_=5.0 V. Horizontal dotted lines in (**d**) are the Landau level filling factors (*ν*) defined as *R*_*xy*_=*h*/*νe*^2^ (*ν*=4, 5, 6).

The best demonstration of the QHE is achieved using a sample which becomes insulating below 1 K on floating the back-gate electrode. The application of a positive *V*_G_ accumulates the charge carriers in the δ-doped region and the device becomes conducting for *V*_G_>4.3 V. Figure 2a,b shows *V*_G_ dependence of carrier density and mobility of the device at 50 mK. As *V*_G_ increases, the carrier density, as determined from the Hall effect measurement, increases from 7.7 × 10^11^ to 1.2 × 10^12^ cm^−2^ (See Supplementary Note 1), roughly following the linear relationship. The slope of 2.8 × 10^11^ cm^−2^ V^−1^ depicted as broken line corresponds to the model of a plane-parallel capacitor assuming a dielectric constant *ɛ*=20,000 for the gate insulator STO^22^ and a substrate thickness of 500 μm. The mobility shows a maximum value of 18,000 cm^2^ V^−1^ s^−1^ at a carrier density of 1.0 × 10^12^ cm^−2^ (see Fig. 2b) and thus is comparable with the value obtained in metallic as-grown devices shown in Fig. 1b. Figure 2c,d shows an example of the *R*_*xx*_ and *R*_*xy*_ at 50 mK for *V*_G_=5.0 V (1.2 × 10^12^ cm^−2^). First, one recognizes instantly the strong oscillations of *R*_*xx*_, whose well-developed *R*_*xx*_ minima coincide with the Hall plateau structures of *R*_*xy*_. Second, the plateaus at the negative field axis can be clearly assigned to Landau level filling factors *ν*=4 and 6 in *R*_*xy*_=*h*/*νe*^2^. At the positive field axis, *R*_*xy*_ is also well quantized for *ν*=4, whereas *ν*=6 deviates from the exact quantized value. Furthermore, Supplementary Note 2 shows that the *R*_*xx*_ minima at integer-filling factors show thermaly activated behaviour. Given the current state-of-the-art for STO heterostructures, of which quality is doubtless lower than that of the well-established high-mobility semiconductor heterostructures^2^, the slight asymmetry in magnetotransport with respect to the magnetic-field direction is not very surprising. Despite all imperfections that the current structure may suffer from, for example, disorder, charge-carrier inhomogeneity, the all metrics mentioned above strongly suggests the realization of QHE in the δ-doped STO.

**Figure 3:**
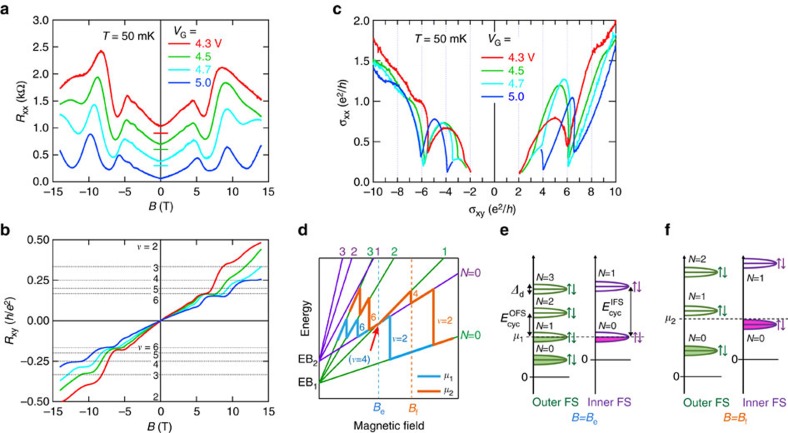
Carrier density dependence of quantum transport in δ-doped SrTiO_3_. (**a**,**b**) Longitudinal resistance *R*_*xx*_ (**a**) and Hall resistance *R*_*xy*_ (**b**) versus magnetic field *B* measured at 50 mK with various *V*_G_'s. For *R*_*xx*_, the traces are shifted vertically for clarity as denoted by horizontal bars. Horizontal dotted lines in **b** are the Landau level filling factors (*ν*=2−6). (**c**) Parametric plots of (*σ*_*xy*_(*B*), σ_*xx*_(*B*)) for various gate voltages at 50 mK. (**d**) A schematic of Landau levels as a function of magnetic field. Green and purple lines correspond to the Landau levels (*N*) of outer (EB_1_) and inner (EB_2_) subbands, respectively. All lines contain up and down spin states, assuming spin degeneracy due to the small *g*-factor. The thick blue and orange lines illustrates the chemical potentials (*μ*_1_ and *μ*_2_) for low and high *V*_G_'s. If the chemical potential (blue line *μ*_1_) is located at the crossing point of *N*=1 of the outer subband and *N*=0 of the inner subband (indicated by the arrow), then *ν*=4 is expected to vanish. Shifting the chemical potential upward away from this crossing point (exemplified by the orange line (*μ*_2_)), *ν*=4 can appear. (**e**) Landau level arrangement when *ν*=4 disappears. Δ_d_ is the Landau level broadening. 
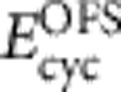
 and 
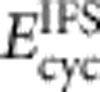
 are the cyclotron energies of the outer and inner FSs, respectively. Note that each Landau level *N* is spin degenerate. (**f**) Landau level arrangement when *ν*=4 can be observed.

To inspect the QHE in more detail, we acquired the magnetoresistance traces of *R*_*xx*_ and *R*_*xy*_ at various *V*_G_'s and display them in Fig. 3. In accordance with expectations, when *V*_G_ increases (that is, the carrier density increases), the positions of valleys and peaks in *R*_*xx*_ (Fig. 3a) systematically shift to higher magnetic fields. However, a distinct behaviour is found for filling factors *ν*=4 and *ν*=6. While the state at *ν*=6 with a well-developed plateau at or close to *R*_*xy*_=*h*/6*e*^2^ (Fig. 3b) and *R*_*xx*_ minima is observed for all *V*_G_'s, the quantum Hall state at *ν*=4 strikingly vanishes, that is, *R*_*xy*_ deviates from the quantized value and *R*_*xx*_ minimum disappears, when *V*_G_ is lowered. To visualize the quantization behaviour, Fig. 3c replots the data in the plane of *σ*_*xx*_ (*B*) and *σ*_*xy*_ (*B*) with *B* being the parameter for various *V*_G_'s. Such representation demonstrates that the curves seem to converge towards (*σ*_*xy*_, *σ*_*xx*_)=(±6*e*^2^/*h*, 0), which seems to be a stable point for all *V*_G_'s, while (±4*e*^2^/*h*, 0) forms only at high *V*_G_ (high carrier density). It should be noted that such conversions can be observed only when *R*_*xy*_ plateau and *R*_*xx*_ minima are realized simultaneously. It is quite evident that the observed quantization is imperfect since the *σ*_*xy*_ deviates from its exact quantization value and *σ*_*xx*_ does not reach zero. Such behaviour might be caused by an additional conduction channel or some final bulk conductance remaining even in the regime of the QHE, which does not show localization behaviour in the magnetic field. We believe that improving the sample quality and gaining more knowledge on the origin for the disorder in the heterostructure (among the suspects is the inactive La dopants) will result eventually in the exact quantization of *σ*_*xy*_ (*νe*^2^/*h*) concomitant with *σ*_*xx*_ reaching zero. Finally, we note that a conspicuous absence of odd filling factors at all *V*_G_'s indicates either the spin degeneracy or the orbital degeneracy at high magnetic fields. Since our density functional theory (DFT) calculation presented below rules in (out) the former (latter), we expect that the Landau levels at high magnetic field are spin degenerate. This expectation may also be valid when the Zeeman spin splitting (*gμ*_B_*B,* where *g* is the electron *g*-factor) is smaller than the other energy scales such as that arising from the disorder.

### Electronic structures of δ-doped SrTiO_3_

**Figure 4:**
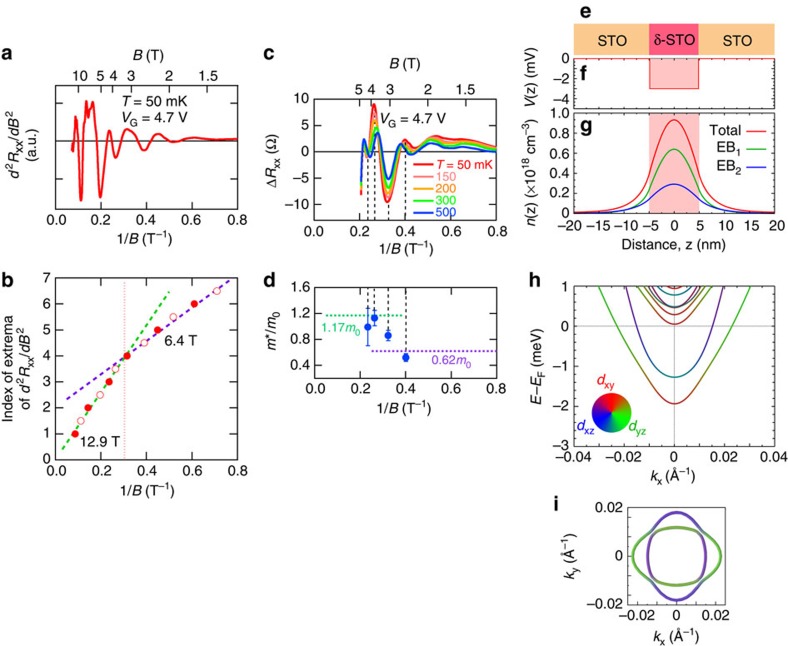
Electronic structures of δ-doped SrTiO_3_. (**a**) *d*^2^*R*_*xx*_/*dB*^2^ as a function of 1/*B* at 50 mK with *V*_G_=4.7 V, corresponding to *n*=1.0 × 10^12^ cm^−2^. (**b**) Indices of extrema in *d*^2^*R*_*xx*_/*dB*^2^ as a function of 1/*B*. The integer (half integer) indices are assigned to the valley (peak) positions of *R*_*xx*_ denoted by the closed (open) circles. The slope changes from 12.9 to 6.4 T around 1/*B*=0.3 T^−1^ (*B*=3.3 T) as denoted by the dotted line. (**c**) SdH oscillations with *V*_G_=4.7 V after subtracting the non-oscillating background, Δ*R*_*xx*_, is plotted as a function of 1/*B* at various temperatures. (**d**) Effective mass m* experimentally determined from the temperature dependence of oscillation amplitude at each Δ*R*_*xx*_ extremum is shown by closed circles and those theoretically derived are indicated by horizontal dotted lines. (**e**–**i**) The calculated band structure of δ-doped STO-QW. The δ-doped region is assumed to be sandwiched between two sufficiently large slabs of undoped STO as shown in **e**. Assuming a square potential for the QW (**f**), the total and subband-decomposed charge-carrier density distributions (denoted by EB_1_ for outer FS and EB_2_ for inner one) are plotted in **g** and **h**. The corresponding orbital-projected band structure of QW. (**i**) The Fermi surface formed by the confined subbands.

To shed light on the mechanism of quantum oscillations, we have calculated the electronic structure of a δ-doped STO thin-film sandwiched between two sufficiently thick undoped STO slabs using realistic tight-binding supercell calculations, incorporating the band-bending potential in the δ-doped region (see Methods). To be consistent with the design of our experimental heterostructure, the thickness of the quantum well (QW) is considered to be 10 nm, as schematically shown in Fig. 4e. We have assumed a square potential well (Fig. 4f) and varied its depth until the total amount of carrier density confined inside, and in the vicinity of QW, becomes *n*=1.0 × 10^12^ cm^−2^. Under these conditions, the QW formed at the δ-doped region confines two subbands. As shown in Fig. 4h,i by the false colour scale, both subbands are dominantly made of heavy *d*_*xz*/*yz*_ orbtials at the Fermi level *E*_*F*_, whereas the *d*_*xy*_ orbital is the main contributor at the bottom of the lowest subband. It is to be noted that these subbands are distinct from the subbands previously observed at the surface of STO and KTaO_3_ (refs 6, 23). In those systems, the near surface band-bending potentials are much deeper but effectively confined within a much narrower region (that is, a few STO units). Consequently, *d*_*xy*_ orbitals contribute dominantly to the lowest subbands thereby making them highly dispersive, whereas the *d*_*xz*/*yz*_ can only contribute to the heavy subbands at much higher energies near the Fermi level. In the present system, on the other hand, due to the large width of the δ-doped STO QW (10 nm), both the in-planar *d*_*xy*_ orbital and out-of-planer *d*_*yz*_/*d*_*xz*_ orbitals can be comparably confined within the QW region, thereby causing each subband to have complicated orbital characters. Moreover, the shallowness of the QW potential (which is due to the low carrier density) combined with the spin–orbit coupling can further complicate the orbital character of the subbands. In fact, the confined carrier density here is so low that the corresponding QW potential is expected not to be deeper than a few meV. This value is much smaller than the energy scale of the spin–orbit coupling between Ti *t*_2g_ states (∼36 meV). Therefore, the resulting subbands below Fermi level are subject to a strong orbital mixing, as shown in Fig. 4.

The resulting Fermi surface (FS) is composed of two spin-degenerated pockets, which are coaxially centred at the Γ point (see Fig. 4i). The outer FS has a star-shaped geometry and encloses an area of *A*_OFS_=0.00138 Å^−2^. The inner FS, on the other hand, has a less distorted shape with an enclosed area of *A*_IFS_=0.00077 Å^−2^, almost half of *A*_OFS_. For both subbands denoted by EB_1_ for the energy band of outer FS and EB_2_ for that of inner FS in Fig. 4g, the corresponding carrier densities are mainly distributed inside the QW. However, they also have a fading tail reaching up to 15 nm beyond the δ-doped region. Using the Onsager relation *F*=(Φ_0_/2π^2^)*A*, where Φ_0_ is the flux quantum, the frequency of SdH oscillations (or the slope of fan diagram) corresponding to outer and inner FS's are found to be 12.7 and 7.1 T, respectively.

The SdH oscillations are also analysed experimentally. To properly determine the positions of peaks and valleys in *R*_*xx*_ for the small amplitude oscillations at low fields, we take *d*^2^*R*_*xx*_/*dB*^2^ and plot it as a function of 1/*B* at *V*_G_=4.7 V (see Fig. 4a). We then assign the integer indices to the *d*^2^*R*_*xx*_/*dB*^2^ peaks (valley positions of *R*_*xx*_), denoted by the closed circles in Fig. 4b and the half integer indices to the *d*^2^*R*_*xx*_/*dB*^2^ valleys (*R*_*xx*_ peaks), indicated by the open circles in Fig. 4b. The frequency is deduced from the slope of indices versus 1/*B*. The change of the slope at 1/*B*=0.3 T^−1^ (*B*=3.3 T) from 12.9 T at high field to 6.4 T at low-field region signals two transport regimes.

## Discussion

These two values agree quite well with the respective band calculation data as shown above. While the fact that the ratio of slope change is close to two may be interpreted as the spin degeneracy lifting at high field, this change turns out to be due to the peculiar subband structure of the present STO 2DES (see Supplementary Note 3). Taking the spin degeneracy into account, the total carrier density extracted from these two SdH frequencies (6.4 and 12.9 T) is found to be 9.0 × 10^11^ cm^−2^ (3.0 × 10^11^ cm^−2^ for 6.4 T and 6.0 × 10^11^ cm^−2^ for 12.9 T); slightly lower than the carrier density estimated from the Hall effect, 1.0 × 10^12^ cm^−2^. This deviation is much smaller than that of previous reports^8^,^16^ and is likely due to a minor contribution from an additional conduction channel as mentioned above, which can only affect the Hall effect (and not the SdH oscillations). This may accordingly explain why *R*_*xx*_ shows non-vanishing values in its minima. For the two peculiar oscillations, we expect that the outer FS contributes to the oscillations at high-magnetic-field region and the inner FS dominates the observed oscillations at low fields. This is due to the fact that at low fields the amplitude of oscillations originating from the high index Landau levels of outer FS are much weaker than that of low index of Landau levels of inner FS. At 3.3 T, the inner FS is expected to reach its quantum limit, and thus can no longer contribute to the oscillatory part of *R*_*xx*_. On the other hand, the outer FS, due to its larger area, is still far from its quantum limit. Therefore, the oscillations observed at higher fields, are merely from the outer FS.

We have also calculated the cyclotron effective mass for each FS (
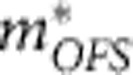
 and 
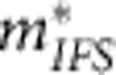
: the cyclotron effective mass for outer and inner FS) using the relation 
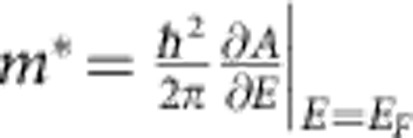
 and obtained 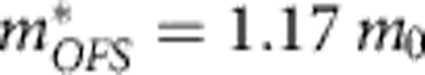 and 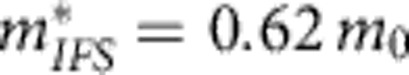. To compare these values with the experiment, we have deduced m* from the temperature dependence of the SdH oscillations after subtracting the non-oscillating background (Δ*R*_*xx*_, Fig. 4c). To be consistent with our calculations, we consider the *R*_*xx*_ oscillations for *n*=1.0 × 10^12^ cm^−2^ corresponding to *V*_G_=4.7 V. m* is then determined at each Δ*R*_*xx*_ extremum, as denoted by the dashed lines in Fig. 4c,d (additional information is provided in the Supplementary Note 4). Because of the weighted contribution of large and small FSs to *R*_*xx*_ oscillations, the electron mass at low fields is clearly smaller than that at high fields despite the uncertainty in estimated values of m* (indicated by the error bars in Fig. 4d). This tendency is in accordance with our calculations of 
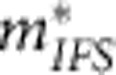
 and 
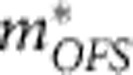
, predicting a smaller (larger) m* for the inner (outer) FS as indicated by horizontal lines.

Such a mixed subband contribution strongly affects the appearance of the QHE. Taking into account the relative positions of the subbands and their different effective masses, a schematic fan diagram for the spin-degenerate Landau levels stemming from each subband is depicted in Fig. 3d; green and purple lines correspond to the Landau levels (*N*) of outer (EB_1_) and inner (EB_2_) subbands, respectively. This diagram can explain both the disappearance of *ν*=4 and the stability of *ν*=6. As pointed out in refs 24, 25, an even integer-filling factor is suppressed if two conditions are fulfilled: odd filling factors should occur simultaneously in each subband and two Landau levels, each from one of the subbands, should be degenerate at the chemical potential *μ*. Figure 3e visualizes a particular arrangement of Landau levels for which the quantum Hall state (QHS) *ν*=4 is suppressed. Here the chemical potential, denoted as *μ*_1_, is located at the crossing between *N*=1 of EB_1_ and *N*=0 of EB_2_. In this situation, EB_1_ is at filling factor *ν*=3 and EB_2_ is at *ν*=1, so that the total filling factor becomes 4. However, this does not lead to the QHE, since *μ*_1_ is not in a gap. Changing the charge-carrier density, and correspondingly relative population of the subbands, can shift the chemical potential into the gap between the Landau levels and thus lead to the formation of QHS at *ν*=4 as illustrated in Fig. 3f. In the same manner, we can explain the stability of QHS at *ν*=6. Considering the fact that EB_1_ and EB_2_ have different energies, the QHS at *ν*=6 can be suppressed if the filling factors of these subbands are *ν*=5 and *ν*=1, respectively. This, however, imposes a large imbalance on the charge-carrier densities of subbands, which cannot practically be realized in our STO structure. Thus, the QHS *ν*=6 is found to be stable in our experiment. In addition, one can find that slightly higher chemical potential than *μ*_2_ makes *ν*=4 and 8 quantum Hall plateau more stable. However, much higher magnetic field will be needed to observe the *ν*=2 plateau in that case.

In conclusion, we have observed QHE in δ-doped STO grown at high temperature by MOMBE. This is the first observation of the QHE in perovskite oxides. The Hall conductance is quantized at even integer-filling factors. The absence of odd integer-filling factors is proposed to be due to the small *g*-factor of electrons. Using sophisticated electronic structure calculations, the peculiar behaviour of the QHE is attributed to the strong orbital anisotropy of subbands in the QW formed at the δ-doped STO region. For *ν*=4, the corresponding plateau disappears at certain low carrier densities due to a crossing of the two subbands. The realization of such a unique QHE system opens up a new route to explore the unknown aspects of quantum transport and their functionalities.

## Methods

### MBE growth

All films were grown by metal organic gas source molecular-beam epitaxy (MOMBE) at a high temperature^13^,^16^,^21^,^26^. In this method, Sr and La flux were evaporated from a conventional effusion cell with a pure elemental source, where La atoms act as dopants by substituting the Sr sites. For the Ti source, Titanium tetra isopropoxide (TTIP) (99.9999 %) was kept around 100 °C for thermal evaporation from a MO container without any carrier gas. Sr flux was kept at a beam equivalent pressure (BEP) of 8 × 10^−8^ torr and TTIP was varied to optimize the TTIP/Sr ratio (see Supplementary Note 5). La flux was controlled by the temperature of the effusion cell calibrated by a quartz crystal microbalance thickness monitor as described in Supplementary Note 6. Although the sheet La concentration in all samples is set to 3 × 10^13^ cm^−2^, the measured sheet carrier density at 2 K varies widely between 1 × 10^12^ and 3 × 10^13^ cm^−2^ as shown in Fig. 1b. Such a variation might be caused by several reasons. As shown in Supplementary Note 6, Supplementary Fig. 6, and Supplementary Fig. 7, one is the experimental uncertainty of actual La beam flux and activation ratio of dopant. In fact, the carrier density at room temperature is found to vary between 1.5 × 10^13^ and 4.5 × 10^13^ cm^−2^. Another reason is a partial freezing of charge carriers while lowering the temperature. We found this freezing is more pronounced for samples with smaller carrier density at room temperature. This expands the variation of carrier density at 2 K towards smaller carrier density side. The reason of partial freezing is not clear but such a behaviour has been commonly observed in a number of previous studies on STO-based 2DESs^8^,^18^. Taking into account the fact that thick single crystalline films with comparable or smaller La concentration do not show such a behaviour^14^, we presume that the charge-carrier freezing is related to the localization of carriers due to disorder effect pronounced by confinement. Distilled pure ozone as oxidizing agent was generated and supplied from MPOG-104A1-R, MEIDENSHA Co. to the chamber at a pressure of 5 × 10^−7^ torr. The films were grown at a substrate temperature of 1,200 °C, which can be achieved with a semiconductor-laser heating system^27^ and is much higher than that used in previous MOMBE^13^,^16^,^21^. Despite such a high-temperature growth, the depth profile measurement of La density revealed that La diffusion is absent as shown in Supplementary Note 7. To fill the oxygen vacancies formed during the growth, the samples were annealed in the growth chamber at 600 °C in *P*_ozone_=1 × 10^−6^ torr for 1 h after deposition. In contrast to previous reports^21^, the growth window of stoichiometric films is much wider, across a BEP ratio of TTIP/Sr=25–140, due to higher growth temperature by laser heating as described in Supplementary Note 5. The lattice constant is constant at 3.905 Å, which is same with that of stoichiometric single crystals. The mobility at low temperatures exceeds 53,000 cm^2^ V^−1^ s^−1^ for the thick homogeneous La-doped STO film, which is higher than the record value of bulk single crystal of STO and comparable to the record of electron doped STO film reported previously^13^.

### Electronic structure calculation

To calculate the interface band structure, we initially performed a DFT calculation using the Perdew–Burke–Ernzerhof exchange-correlation functional, modified by Becke–Johnson potential as implemented in the WIEN2K program^28^. Relativistic effects, including spin–orbit coupling, were fully included. The muffin–tin radius of each atom *R*_MT_ was chosen such that its product with the maximum modulus of reciprocal vectors *K*_max_ become *R*_MT_*K*_max_=7.0. The Brillouin zone was sampled by a 15 × 15 × 15 *k*-mesh. The resulting DFT Hamiltonian was then down-folded using maximally localized Wannier functions^29^,^30^,^31^ to generate a 200 unit cell tight binding supercell stacking along [001] direction with additional on-site terms, accounting for the QW potential. The same method has been already applied and successfully reproduced the results of ARPES data of the 2DEG confined at the surface of STO^6^. Assuming a 10-nm-thick interface, the depth of QW was varied until the total amount of carrier density confined at, and in the vicinity of, the interface became *n*=1.0 × 10^12^ cm^−2^.

We emphasize that in our tight-binding supercell Hamiltonian, there is no adjustable parameter other than an on-site potential term, representing the band-bending potential in the QW. Even for this bending potential we consider the same width as that realized in our experiment. The only variable parameter in our calculation, as mentioned above, is the depth of the potential that is chosen such that it yields the same confined charge-carrier density as that observed in our experiment (1 × 10^12^ cm^−2^).

### Data availability

The authors declare that the data supporting the findings of this study are available within the article and its Supplementary Information.

## Additional information

**How to cite this article:** Matsubara, Y. *et al*. Observation of the quantum Hall effect in δ-doped SrTiO_3_. *Nat. Commun.* 7:11631 doi: 10.1038/ncomms11631 (2016).

## Supplementary Material

Supplementary Information Supplementary Figures 1-8, Supplementary Notes 1-7 and Supplementary References
