# Supplementary material for: Observation of the quantum Hall effect in δ-doped SrTiO3
Source: Nat Commun. 2016 May 27;7:11631. doi: 10.1038/ncomms11631 (PMC4894966; doi:10.1038/ncomms11631)
Supplement: Supplementary Information — Supplementary Figures 1-8, Supplementary Notes 1-7 and Supplementary References [file ncomms11631-s1.pdf]

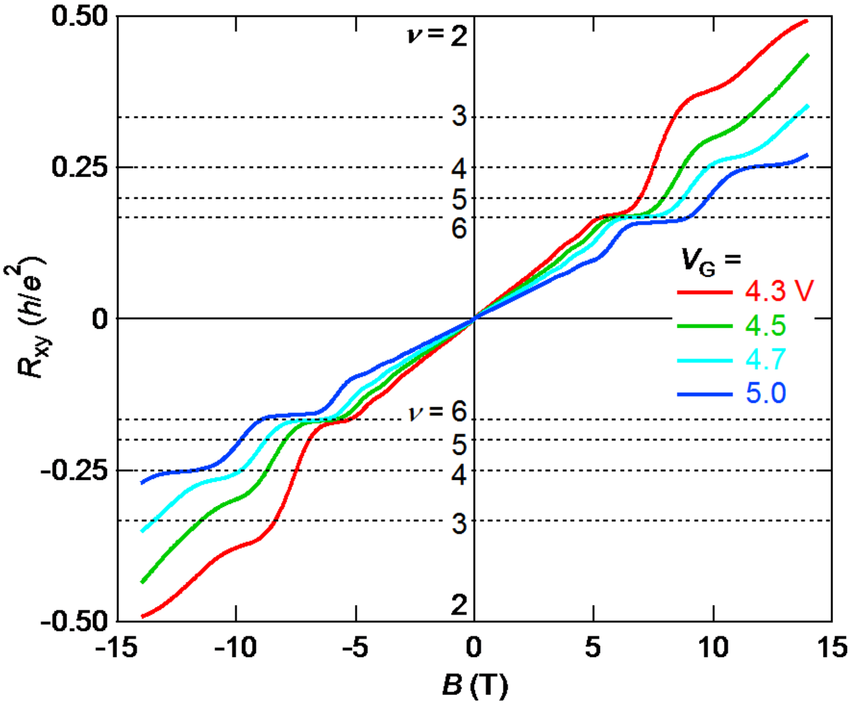

Supplementary Figure 1 Hall resistance R_{xy} versus magnetic field measured at 50 mK with various V_G's after the subtraction of B-symmetric component in R_{xy}.

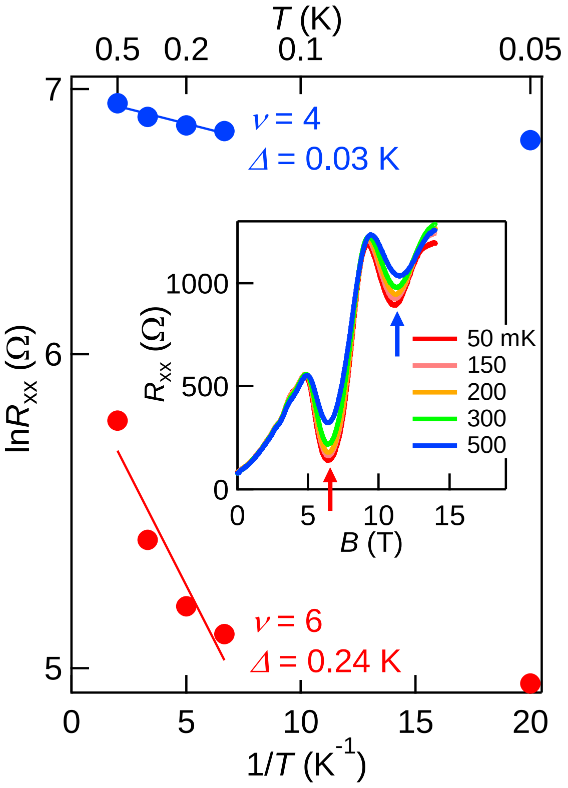

Supplementary Figure 2 Arrhenius plots of the resistance minima for $\nu = 6$ (at $B = 6.7$ T) and $\nu = 4$ (at $B = 11.2$ T) for $V_G = 4.7$ V to estimate the activation energy. The values are evaluated by the fitting lines between 150 mK and 500 mK. The inset shows the R_{xx} as a function of magnetic field for $V_G = 4.7$ V at various temperatures. The arrows indicate the resistance minima which are analyzed.

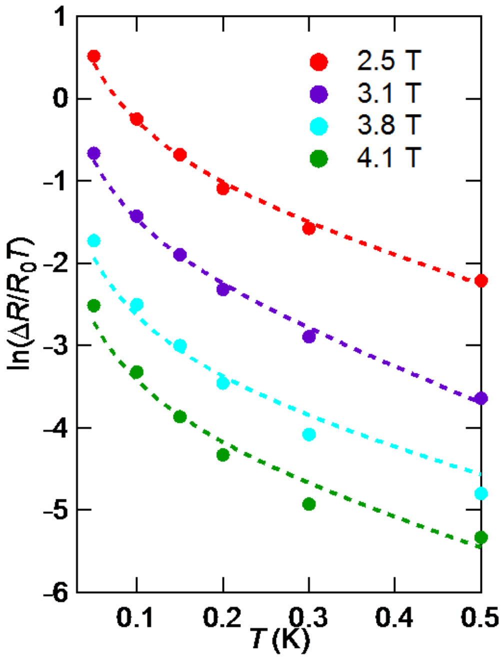

Supplementary Figure 3 Temperature dependence of $\ln(\Delta R/R_0T)$ for the oscillation peak at 2.5 T, 3.1 T, 3.8 T, and 4.1 T where R_0 denotes R_{xx} at B = 0 T. To estimate m^*, the data is fitted with the dotted curve. The curves are vertically shifted for clarity.

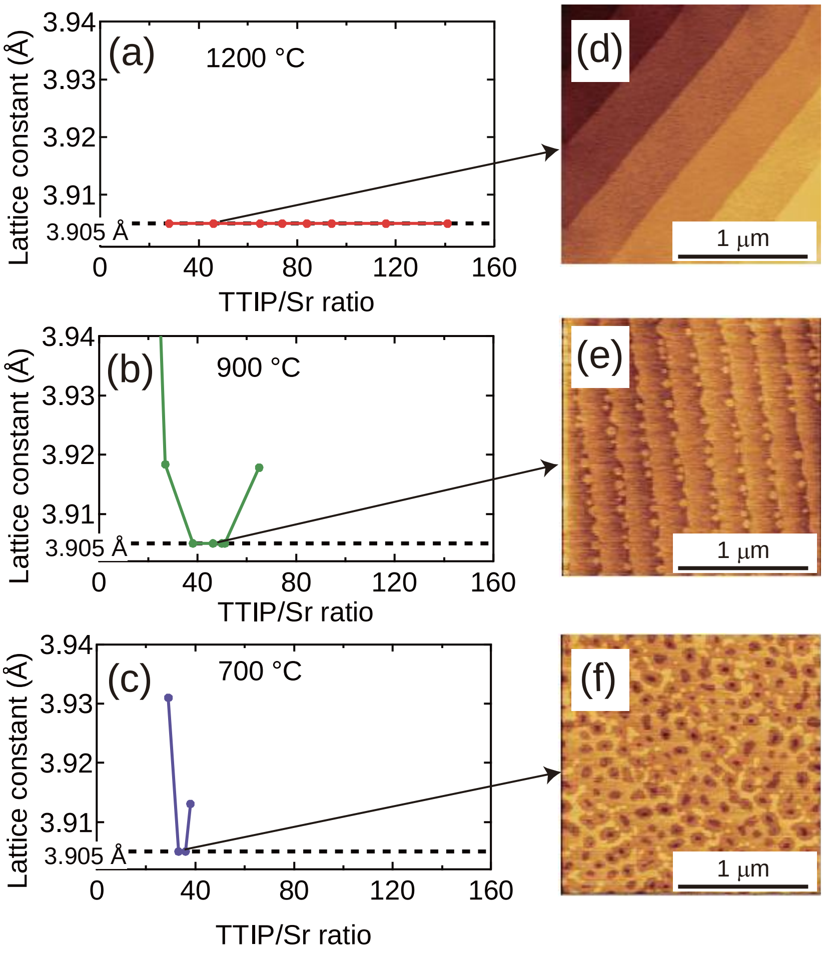

Supplementary Figure 4 Out of plane lattice constant as a function of TTIP/Sr ratio grown at (a) 1200°C, (b) 900°C (c) 700°C. AFM images (d)-(f) are shown for the film grown at a certain TTIP/Sr ratio denoted by arrows for each growth temperature.

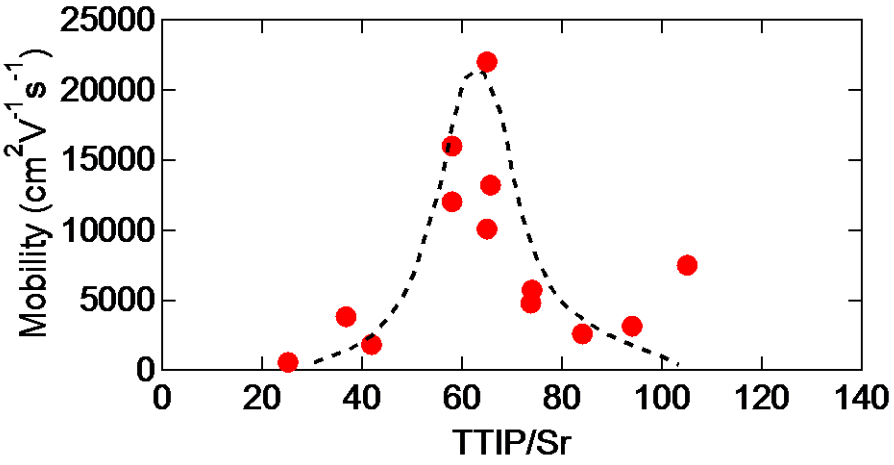

Supplementary Figure 5 Electron mobility at 2 K for δ-doped STO structures grown at 1200°C as a function of TTIP/Sr ratio. Dotted line is a guide to the eyes.

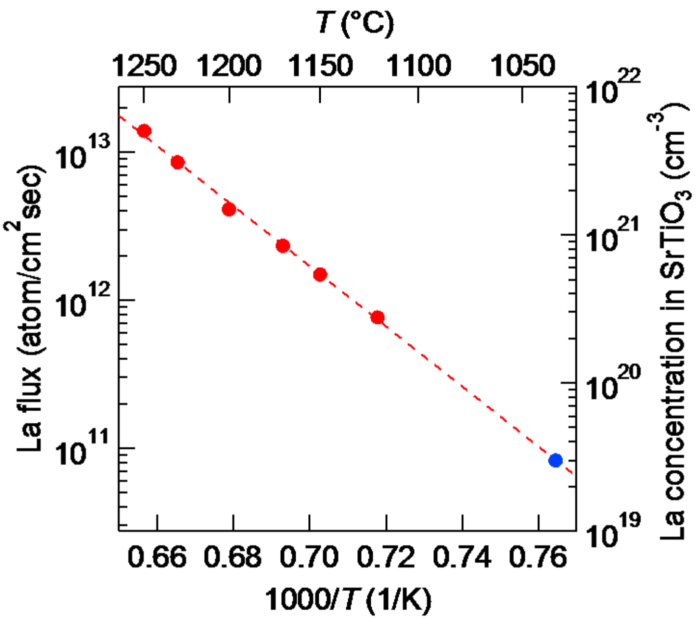

Supplementary Figure 6 La flux (red circles) determined by a quartz crystal microbalance as a function of 1000/*T*. The right axis indicates corresponding La doping concentration in the La doped STO films. For the La doped layer in this study, we employed the flux condition denoted by the blue circle.

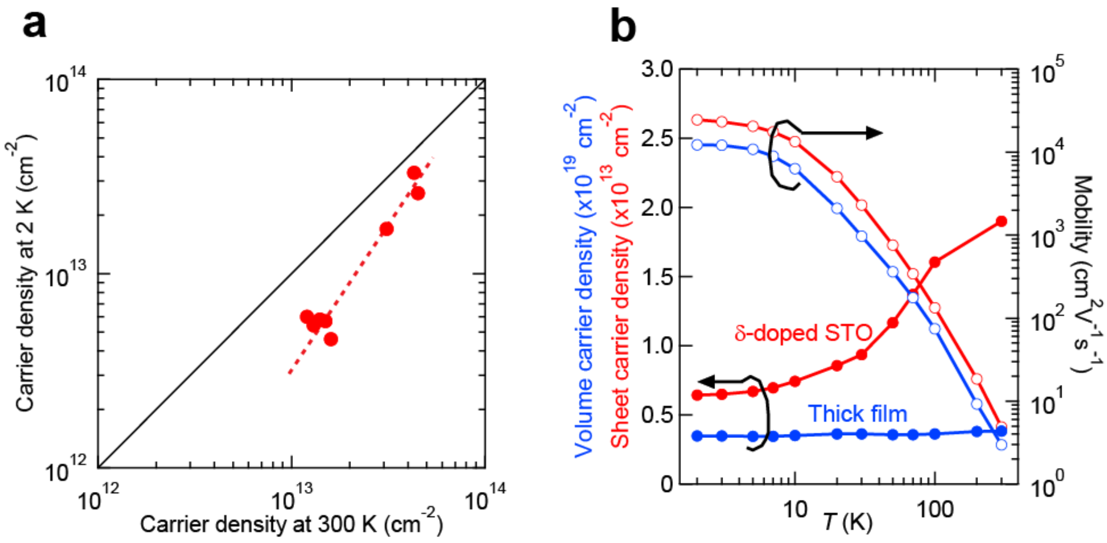

Supplementary Figure 7 (a) Relationship of carrier density at 300 K and that at 2 K. Broken red line is a guide to eyes. (b) Carrier density and mobility as a function of temperature for a typical δ-doped STO sample (red) and a thick La-doped STO film (blue).

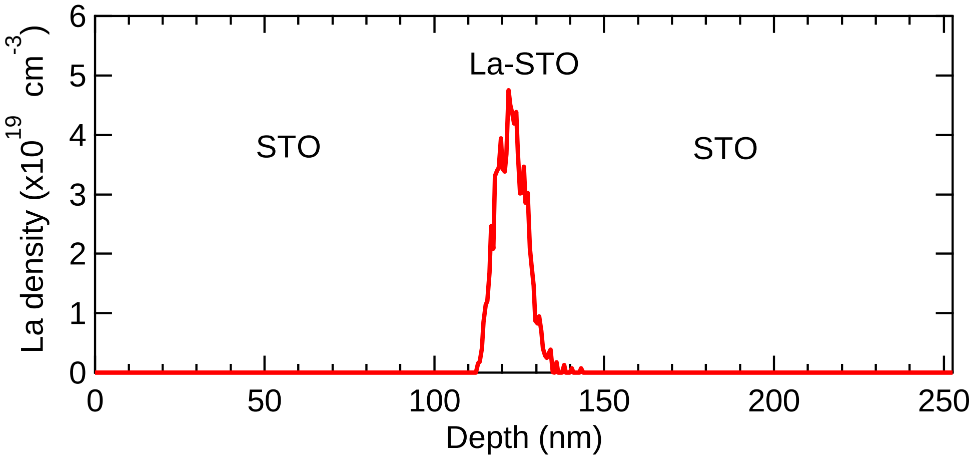

Supplementary Figure 8 The depth profile of La density measured by a secondary ion mass spectroscopy for a STO/La doped STO/STO quantum well structure fabricated at 1200°C. The La density is calibrated by a bulk single crystal of La doped STO (La: 1.7×10^{19} cm^{-3}) as a reference.

Supplementary Note 1 | Estimation of carrier density from the Hall effect measurement

In Fig. 2c, 2d, 3a, and 3b, one can find an asymmetry in R_{xx} and R_{xy} with respect to the magnetic field direction which is seen in quantum transport measurements of many low dimensional systems, even in the extreme high mobility GaAs heterostructures [1]. Even a small inhomogeneity damps the amplitude of the oscillations. The fact, that the amplitude of SdH in our sample is different for positive and negative field polarity, means that the charge carrier density inhomogeneity plays a role. Another cause for the asymmetry might be the van der Pauw configuration in sub millimeter scale. For precise estimation, the carrier density in Fig. 2a is calculated from the slope of R_{xy} (B) between -2 T and 2 T after its symmetrization as shown in Supplementary Fig. 1.

Supplementary Note 2 | Activation behaviors of quantum Hall states

To determine the activation energies (Δ) of quantum Hall states, the temperature dependence of resistance is analyzed for V_G = 4.7 V which is shown in the inset of Supplementary Fig. 2. Supplementary Figure 2 shows the Arrhenius plots of the resistance minima for ν = 6 (at B = 6.7 T) and ν = 4 (at B = 11.2 T) denoted by arrows in the inset. We determine the value of Δ according to the formula $R_{xx} = R_0\exp(-\Delta/2k_BT)$, where k_B is the Boltzmann constant. Except for lowest temperature data points at 50 mK, the data obey the activation formula. The large difference of activation energy for two minima indicates that ν = 6 state is almost quantized while the ν = 4 state suffers from a delocalization.

Supplementary Note 3 | Possibility of another interpretation for Landau level formation with spin split bands

The present study concludes that the presence of two Fermi surface is important for the observed phenomena. The assumption of a single Fermi surface with a Zeeman splitting in the magnetic field is inconsistent with the observation of a sequence of only even filling factors. To make this apparent, here we consider following two scenarios from general point of view for 2DES Landau level formation assuming a single Fermi surface. Case (1) : If the Zeeman energy (E_Z) is large enough, e.g. $E_z > h\omega_c/4\pi$ and larger than the disorder broadening, we should be observing a sequence of odd and even integer filling factors without any skipping (1, 2, 3, 4, 5, 6, …), which is not the case in our experiment. Case (2): If the E_z is comparable with $h\omega_c/2\pi$, the spin-up and spin-down states of adjacent Landau levels may overlap, similar as it is seen in ZnO [2-4] and therefore one would expect observing a sequence of odd filling factors (1, 3, 5, 7, …), which is also not the case in the current experiment.

Our model suggests that the change of the SdH frequency by factor 2 shown in Fig. 4b is the signature of the change in the contribution of two subbands to the magnetotransport, rather than the result of Zeeman splitting. Although we do not have enough experimental supportive facts which may point to the importance of the Zeeman splitting, we do not completely exclude the role of the Zeeman energy in our structures. The case of two subbands model with a significant Zeeman splitting, i.e. lifted spin degeneracy of each Landau level, will result in the formation of four levels with the same Landau level index. The relative arrangement of these levels on the energy axis will depend on both the quantum well confinement potential and the relation between the cyclotron and the Zeeman energies. The test of such a potential scenario will require both the samples with a much improved quality, i.e. low disorder, and the application of higher magnetic fields.

Supplementary Note 4 | Estimation of effective mass m^* under various magnetic fields

Temperature dependence of the SdH oscillation amplitude (ΔR) is described by $\Delta R/R_0 = 4\chi\exp(-\pi/\omega_c\tau_q)/\sinh\chi$, where R_0 is the resistance at $B = 0$ T, $\chi = 4\pi^3 k_B T/h\omega_c$, $\omega_c = eB/m^*$ is the cyclotron frequency, and τ_q is the quantum scattering time [5]. Supplementary Figure 3 depicts $\ln(\Delta R/R_0 T)$ versus T for the oscillation amplitude at various magnetic fields with fitting curves (dotted line). By this fitting, the electron effective mass is deduced and each m^* value was plotted in Fig. 4d in main text.

Supplementary Note 5 | TTIP/Sr ratio dependence of the mobility at low temperatures

Crystal quality and mobility in electron doped STO strongly depends on the stoichiometry, that is, the Sr/Ti ratio [6]. The *c*-axis lattice constant of nonstoichiometric homoepitaxial STO is known to be expanded [7]. In the case of MOMBE growth, a wide growth window, where the *c*-axis lattice constant of the films is independent of the beam flux ratio of TTIP to Sr and is identical with that of bulk STO crystal (0.3905 nm), has been found in previous reports [8]. In this study, a TTIP/Sr ratio was optimized not only by the structure analysis but also by the transport properties for the δ-doped structures. In order to obtain high quality δ-doped structure, high temperature growth using laser heating system was employed [9]. Supplementary Figure 4 shows the out of plane lattice constants of the films grown at various temperatures as a function of TTIP/Sr ratio. AFM images are also given for the films grown at a certain TTIP/Sr ratio inside the growth window (denoted by arrows). As increasing the growth temperature, the growth window of TTIP/Sr ratio becomes wider. In AFM images, though step-and-terrace surface structures are observed for all films in the growth windows, the step edges become dramatically smoother at higher growth temperature. Note that all films grown at 1200°C within a very wide range of TTIP/Sr ratio (from 25 to 140) turned out to have identical lattice constant to that of the substrate. This flux ratio window is much wider than that reported previously for lower substrate temperatures [8].

Though all films grown at 1200°C are identical from the view point of XRD measurement and AFM surface topography measurement, we found a significant dependence of mobility at 2 K on the TTIP/Sr ratio for δ-doped structures. Supplementary Figure 5 shows the mobility of confined 2DES as a function of TTIP/Sr. Around TTIP/Sr=60, a sharp peak structure is seen. We consider that the really optimized condition, which could not be identified by XRD measurement, is the flux ratio at around 60, resulting in maximum of the mobility. With such optimized growth condition, the

devices show higher mobility than 20,000 $cm^2V^{-1}s^{-1}$.

Supplementary Note 6| La flux control

Since we need to control La concentration to very low level, La flux could not be directly monitored by a beam flux monitor (quartz crystal microbalance). Supplementary Figure 6 shows the relation between measured La flux and effusion cell temperature (red circles). The relation can be reasonably fitted with a linear line as Antoine equation. In this study, we controlled the La doping concentration at 3×10^{19} cm^{-3} with setting the cell temperature at 1035°C from the extrapolation of fitting line. The blue circle denotes the flux and the cell temperature used in main manuscript.

Supplementary Figure 7a shows the relation of carrier density at room temperature and that at 2 K. The former varies between 1.5×10^{13} and 4.5×10^{13} cm^{-2}. If we assume that all dopants are activated at room temperature, this variation should be attributed to the variation in actual La concentration. Then, this corresponds to the variation in La cell temperature of about 50°C around 1035°C referring the calibration curve in Supplementary Fig. 6. However, this uncertainty in temperature may be too large. Therefore, we presume that there is uncontrollable variation both in La flux and in activation ratio of dopants at room temperature. Supplementary Figure 7b shows the temperature dependence of carrier density for a δ-doped STO sample and a thick La-doped STO film. All the δ-doped STO samples show such a carrier freezing behavior. As shown in Supplementary Fig. 7a, there is a clear superlinear trend, indicating carrier freezing is more pronounced for the samples with small carrier density at room temperature.

Supplementary Note 7 | La density depth profile in δ-doped STO

To examine the possible diffusion of La ions in the δ-doped STO, the depth profile of La density was measured by a secondary ion mass spectroscopy (SIMS). The sample was prepared under the same growth conditions with a similar structure: a 120-nm-thick bottom STO buffer layer, a 12-nm-thick STO doped with ‘nominal’ 3×10^{19} cm^{-3} La, and a 120-nm-thick top STO capping layer grown on STO single-crystal substrate. As shown in Supplementary Fig. 8, the SIMS measurement reveals that we can judge the appreciable La diffusion is absent, taking into account a spatial resolution of about 5 nm.

Supplementary References

1. Ponomarenko, L. A. *et al.* The effect of carrier density gradients on magnetotransport data measured in Hall bar geometry, *Solid State Communications* **130**, 705–710 (2004).

2. Tsukazaki, A. *et al.* Spin susceptibility and effective mass of two-dimensional electrons in $Mg_xZn_{1-x}O/ZnO$ heterostructures, *Phys. Rev. B* **78**, 233308 (2008).

3. Kozuka, Y. *et al.* Single-valley quantum Hall ferromagnet in a dilute $Mg_xZn_{1-x}O/ZnO$ strongly correlated two-dimensional electron system, *Phys. Rev. B* **85**, 075302 (2012).

4. Maryenko, D., Falson, J., Kozuka, Y., Tsukazaki, A. & Kawasaki, M. Polarization-dependent Landau level crossing in a two-dimensional electron system in a MgZnO/ZnO heterostructure, *Phys. Rev. B* **90**, 245303 (2014).

5. Smrčka, L., Havlova, H. and Isihara, A. Quantum oscillations in two-dimensional systems with finite width, *J. Phys. C* **19**, L475 (1986).

6. Kozuka, Y., Hikita, Y., Bell, C. and Hwang, H. Y. Dramatic mobility enhancements in doped $SrTiO_3$ thin films by defect management, *Appl. Phys. Lett.* **97**, 012107 (2010).

7. Ohnishi, T., Shibuya, K., Yamamoto, T. and Lippmaa, M. Defects and transport in complex oxide thin films, *J. Appl. Phys.* **103**, 103703 (2008).

8. Jalan, B., Moetakef, P. and Stemmer, S. Molecular beam epitaxy of $SrTiO_3$ with a growth window, *Appl. Phys. Lett.* **95**, 032906 (2009).

9. Ohashi, S., Lippmaa, M., Nakagawa, N., Nagasawa, H., Koinuma, H. and Kawasaki, M. Compact laser molecular beam epitaxy system using laser heating of substrate for oxide film growth, *Rev. Sci. Inst.* **70**, 178–183 (1999).
